# mSphere of Influence: Surface Sensing in Biofilm Formation

**DOI:** 10.1128/mSphere.00369-21

**Published:** 2021-05-12

**Authors:** Kara B. De Leόn

**Affiliations:** a Department of Microbiology and Plant Biology, University of Oklahoma, Norman, Oklahoma, USA

**Keywords:** biofilm, surface sensing, subsurface, *Pseudomonas*, cAMP, attachment, biofilms, cyclic AMP

## Abstract

Kara B. De Leόn works in the field of microbial ecology, environmental biofilms, and microbial genetics. In this mSphere of Influence article, she reflects on how the paper “Multigenerational memory and adaptive adhesion in early bacterial biofilm communities” by C. K. Lee et al. (C. K. Lee, J. de Anda, A. E. Baker, R. R. Bennett, et al., Proc Natl Acad Sci U S A 115:4471–4476, 2018, https://dx.doi.org/10.1073/pnas.1720071115) made an impact on her by changing the way she thinks about initial cell attachment to a surface in an environment.

## Abstract (precis)

Kara B. De Leόn works in the field of microbial ecology, environmental biofilms, and microbial genetics.

## COMMENTARY

Microbes attached to a surface as a biofilm are ubiquitous in nature and are medically, industrially, and environmentally important. Growth as a biofilm is evolutionarily advantageous as it gives some stability in a fluctuating environment and provides protection from a variety of environmental challenges, including UV, acid, dehydration, salinity, metal toxicity, phagocytosis, and several antimicrobial agents (reviewed in reference 1). Determining the mechanism by which cells form biofilms is essential for finding ways to prevent biofilms in undesirable places. This knowledge is also critical for understanding how cells survive and at times thrive in harsh environments. Biofilm formation is initiated by the attachment of a cell to a solid surface, but this initial attachment is often reversible. Over time, cells transition from a reversibly attached to an irreversibly attached state at which point cell division and additional cell attachments form microcolonies that grow to form mature biofilms (2). Though biofilms have been studied in depth for a few decades, questions that remain to be answered completely are: how does a cell “know” it is on a surface and what signals the cell to transition from reversible to irreversible attachment? A collaboration between the labs of Gerard C. L. Wong and George A. O’Toole has made great strides addressing both questions (3, 4). In their paper titled “Multigenerational memory and adaptive adhesion in early bacterial biofilm communities,” their teams show how Pseudomonas aeruginosa cells accumulate cyclic AMP (cAMP) as they transiently attach to a surface, and these concentrations provide a “memory” of the surface that is a key step in ultimately transitioning to irreversible attachment (4). Furthermore, this memory spans generations such that the population has a collective and long-lasting memory of the surface (4).

By using single-cell tracking and a reporter system for cAMP, the authors showed that that the majority (∼95%) of “surface-naive” cells only transiently attached to the surface and cAMP remained low. However, following ∼22 h of growth in the culture and transient attachment to the surface, there began to be a marked increase in the number of cells that remained attached to the surface. Planktonic cells were harvested from one flow cell and contained both “surface-naive” and “surface-sentient” cells. It was hypothesized that if there was not memory of surface attachment, there again should be an approximately 22-h lag in attachment when provided a new surface. Interestingly, when these cells were passed into a new flow cell, the lag in attachment was short. By tracking cell lineages, the authors found that cAMP levels were increased in cells that had previously attached to the surface. P. aeruginosa replicated at least once per hour under the conditions of the experiment and the signal (cAMP) and the response (type IV pili) oscillations were offset by 5 h; thus, this was determined to be a multigenerational signal. The authors showed that this “memory” decreased over time when in planktonic incubation and returned to a “surface-naive”-like state after approximately 37 h of planktonic growth.

This study showed that P. aeruginosa cells have a way to measure the stability of a surface in an environment. Frequent contact with the surface led to increased cAMP levels within the cells, and this signal spanned multiple generations. This was an important contribution for understanding that cells sense the surface, and repeated interactions take place across generations before the cells commit to irreversible attachment. While we often think about biofilm as a preferred mode of growth for microbes, they do not seem to make the decision lightly to transition to this state of growth. In the case of P. aeruginosa, the original cell that attaches to the surface is multiple generations away from the cell that ultimately commits to biofilm formation. This has changed how I think about biofilm formation in the environment. Much of my research career thus far has been spent working on microbial community structure and function in subsurface environments. Because the active microbial populations in the subsurface are often found in the attached community (5–7), understanding subsurface biofilms is paramount to understanding the microbial community within this environment. This paper altered my thinking about the initial attachment and biofilm strategies that may be occurring. More studies are needed to determine whether these findings are similar in other organisms and how this memory would function in a fluctuating and open environment. Which state (i.e., surface naive or surface sentient) is most reflected in the environment? If cells are reluctant to commit to a surface, that conflicts with the ecological principle of founder effect where the first to reach a surface wins. The first to reach a surface may not choose to stay. It is intriguing to think that maybe this is a mechanism by which cells “decide” when and where to attach to sediment particles. cAMP is a well-studied signaling pathway that responds to environmental changes, including nutrient availability (8). It is likely that environmental stimuli contribute to intracellular cAMP levels, and if a cAMP “memory” mechanism is present in environmental microbes, increased concentrations might stimulate cell attachment in stable environments with available nutrients.

Furthermore, that this signaling is multigenerational brings about questions as to the strategy cells may be using during cell attachment to a surface. After division, one of the daughter cells was shown to leave the surface the majority of the time (4). Once cAMP levels increased and the culture became “surface sentient,” both daughter cells typically remained attached. Thus, over time, more of the planktonic population and their progeny were likely to remain attached when they contacted a surface. This may be a mechanism by which the population can have a concerted effort to populate a large surface area rapidly and nearly simultaneously once it has been deemed stable. Alternatively, this could be a way in which cells could mitigate resource competition between two daughter cells in an oligotrophic environment. We generally think of early biofilms as forming microcolonies of cells, but this study has shown that during the reversible stage of cell attachment prior to microcolony formation, P. aeruginosa cells prefer to spatially separate from progeny. A subsequent study by these authors has shown that approximately 90% of cells that detached postdivision had the mature flagellum inherited from its ancestor, while the other cell had to form a new flagellum postdivision (9). This strategy may be different with different organisms and warrants further study. A comparison of two P. aeruginosa strains (PAO1 and PA14) has shown similar initial reversible attachment behaviors but differences in preference to commit quickly to surface growth (9). The work by the O’Toole and Wong labs has greatly expanded our understanding of surface sensing and reversible attachment. My lab is now tracking single cells during initial cell attachment and biofilm formation by our sulfate-reducing bacteria. I am excited to see future efforts expanding our mechanistic understanding of biofilm formation in other bacterial lineages and whether these mechanisms are different in environmental, multispecies biofilms.
